# Total Ankle Replacement for Treatment of End-Stage Osteoarthritis in Elderly Patients

**DOI:** 10.1155/2012/345237

**Published:** 2012-06-05

**Authors:** Beat Hintermann, Markus Knupp, Lukas Zwicky, Alexej Barg

**Affiliations:** Clinic of Orthopaedic Surgery, Kantonsspital Liestal, 4410 Liestal, Switzerland

## Abstract

End-stage osteoarthritis of the ankle is a disabling problem, particularly in elderly patients who experience an overall loss of mobility and functional impairment and who then need compensatory adaption. Ankle arthrodesis, which has been demonstrated to provide postoperative pain relief and hindfoot stability, leaves the patient with a stiff foot and gait changes. For elderly patient, these changes may be more critical than generally believed. Additionally, the long duration of healing and rehabilitation process needed for ankle arthrodesis may be problematic in the elderly. In contrast to ankle arthrodesis, total ankle replacement has significant advantages including a less strenuous postoperative rehabilitation and preservation of ankle motion which supports physiological gait. Recently, total ankle replacement has evolved as a safe surgical treatment in patients with end-stage ankle osteoarthritis with reliable mid- to long-term results. Total ankle replacement needs less immobilization than arthrodesis and does allow for early weight-bearing and should be considered as a treatment option of first choice in many elderly patients with end-stage osteoarthritis of the ankle, especially in elderly patients with lower expectations and physical demands.

## 1. Introduction

The ankle joint has a much lower incidence of symptomatic osteoarthritis (OA) compared to other major joints of the lower extremity [1]. This, despite the facts that the articular cartilage in the ankle experiences the greatest contact force per unit area of any major joint in the body and the ankle joint is one of the most commonly injured areas in orthopaedic surgery [1–3]. However, degenerative OA of the ankle is a constantly growing problem: currently approximately 1% of the world's adult population is affected by ankle OA leading to significant mental and physical disabilities [4].

Trauma is the primary cause of ankle OA [5, 6]. Valderrabano et al. reviewed 406 ankles that presented with symptomatic end-stage ankle arthritis and found 78% secondary to previous trauma [6]. Patients usually presented with a lower leg fracture in the history, but also repetitive ligamentous injuries of the hindfoot complex may lead to degenerative OA of the ankle [7]. Primary arthritis (possibly secondary to misalignment) occurred in up to 10% of all patients. The remaining 10–15% of patients developed secondary ankle OA due to the following underlying diseases: rheumatoid disease, hemochromatosis [8], haemophilia [9], gout [10], clubfoot [11], aseptic talar necrosis, and after joint infection.

Ankle arthrodesis remains an important treatment option in patients with end-stage ankle OA [12, 13]. After a successful fusion, patients consistently report both, pain relief and improved mobility [12]. However, many clinical studies describe short- and long-term problems following ankle arthrodesis including acute or chronic infection, delayed union, and decreased functional ability [12, 14]. For the majority of patients who achieve full healing of the arthrodesis, the time of convalescence can be difficult: time duration to achieve complete bone healing may range from 12 to 20 weeks. The postoperative recovery involves some form of immobilization and restricted weight-bearing activities, which can cause significant leg muscle atrophy. Even after the ankle arthrodesis is fully healed, some patients may develop profound dysfunction in the long term. Many authors make note of significant limitations with walking inclines, accommodating uneven ground, driving, and athletic activities [14–16]. Formal gait studies after ankle arthrodesis show decreased cadence and stride with decreased motion of the midfoot and hindfoot complexes [17–19]. Gait and function may be also significantly affected if patients develop adjacent joint OA. Coester et al. found in their long-term clinical observational study that majority of patients who underwent an ankle arthrodesis has developed degenerative changes in the ipsilateral foot but not the knee [20]. Similar findings were observed in the long-term study by Fuchs et al., showing deficits in the functional outcome, limitation in the activities of daily living, and radiological changes in the adjacent joints in patients 20 years after ankle arthrodesis [16].

Although ankle arthrodesis is a valid treatment option for end-stage ankle arthritis, its risks and sequelae cannot be ignored. Total ankle replacement (TAR) using current prosthesis designs have evolved to reliable treatment option in patients with end-stage ankle OA. Therefore, ankle fusion is no longer the “gold standard” therapy in this patient cohort [21]. Despite significant progress, concerns still persist related to the feasibility of TAR in patients with bad bone and soft tissue quality, as is often the case in elderly after previous trauma or systemic disease.

The purpose of this paper is therefore to evaluate the potential benefits of TAR in elderly patients with age over 60 years [22], in particular to its advantages with regard to ankle arthrodesis.

## 2. Biomechanics and Gait Analysis

The biomechanics of gait in healthy patients with nonarthritic ankles are clearly different when compared with patients with arthritic, fused, and replaced ankles [19, 21, 23–25].

In choosing between fusion and TAR, benefits in favour of TAR include restoring ankle motion, improving gait biomechanics, and avoiding advanced adjacent joint degeneration more commonly seen following ankle fusion [19, 21, 23–28]. Restoring or at least improving upon gait biomechanics of patients with end-stage ankle arthritis is one of the main goals of surgical treatment for this disease.

Ankle fusion and TAR patients can be expected to have slower gait velocities when compared with healthy control groups but faster speeds when compared with their preoperative arthritic ankle condition [17, 19, 23, 25]. TAR patients exhibit a fairly symmetric gait, while ankle fusion patients require significant compensatory mechanisms to obtain a steady, symmetric gait, including increased midfoot joint motion as well as increased range of motion of the ipsilateral knee [17, 19, 23, 25].

In summary, though patients with a fused ankle can be expected to have a reasonably efficient gait, TAR may offer the patient a more normal gait with less negative impact on segmental motion of the whole lower limb and stress concentration on adjacent joints.

## 3. Surgical Technique and Postoperative Care

Meticulous preoperative planning is the main step for success of TAR [29]. Evaluation in the outpatient clinic entails a detailed history taking, including an evaluation of previous infection, trauma, surgeries, failure or success of treatments, location of pain, social circumstances, previous and current activity level, expectations of treatment, tolerance for revision surgery and general health, especially as it relates to a history of neuropathy and/or diabetes. Also, all previous medical reports (e.g., surgery reports) and imaging studies should be completely collected.

The routine physical examination includes careful inspection of the entire foot and ankle. Hindfoot stability should be assessed manually with the patient sitting. Ankle alignment and range of motion are assessed with the patient standing. Range of motion is determined clinically with a goniometer placed along the lateral border of the leg and foot [30, 31]. Assessment of the subtalar motion and palpation of sinus tarsi may help to exclude subtalar OA. The patients gait is observed clinically and then analyzed using pedobarography in most patients [32]. All affected ankles need to be preoperatively evaluated based on weight-bearing radiographs in three planes. The Saltzman view is used for standardized assessment of varus and valgus deformity of the hindfoot [33]. Single-photon emission-computed tomography (SPECT-CT) can be performed for an accurate assessment and localization of degenerative changes in the adjacent joints [34, 35].

**Figure 1:**
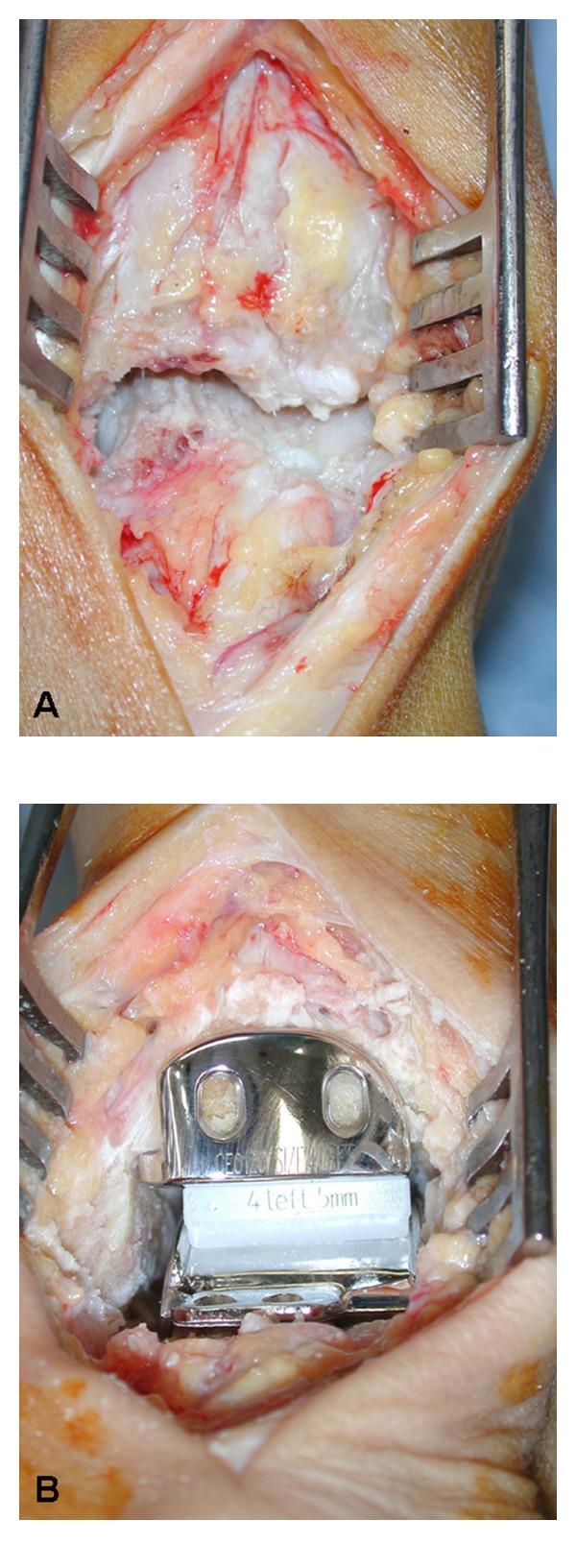
Intraoperative pictures of (A) exposure of arthritic ankle, and (B) after implantation of prosthesis. 66-year-old female patient after septic arthritis.

Most manufactures of ankle prostheses provide reliable instrumentation to perform the appropriate bone cuts and to prepare the resection surfaces to accommodate the prosthesis components. Most surgeons use an anterior approach for exposure of the ankle (Figure 1). Careful dissection of soft tissues and avoidance of any unnecessary soft tissue retraction are keys to success to avoid postoperative wound healing complications. Release of any soft tissue contracture is mandatory to gain joint motion, but also to balance the talus properly within the ankle mortise. Heel cord lengthening may be advised in some cases of equines contracture; its use should be very restrictive as patients will often complain about longstanding soft tissue pain along the tendon and loss of plantar flexion power.

Combined peritalar and ankle arthritis, and complex misalignment of the ankle joint complex are complex and challenging clinical entities [36–40]. Combined peritalar and ankle arthritis and varus/valgus preoperative deformity can be successfully treated with TAR in selected cases but need in most instances additional procedures at the same time [39]. Attention to detail, a meticulous preoperative evaluation, and a carefully planned or staged surgery optimize the chances of a successful result [39, 41].

**Figure 2:**
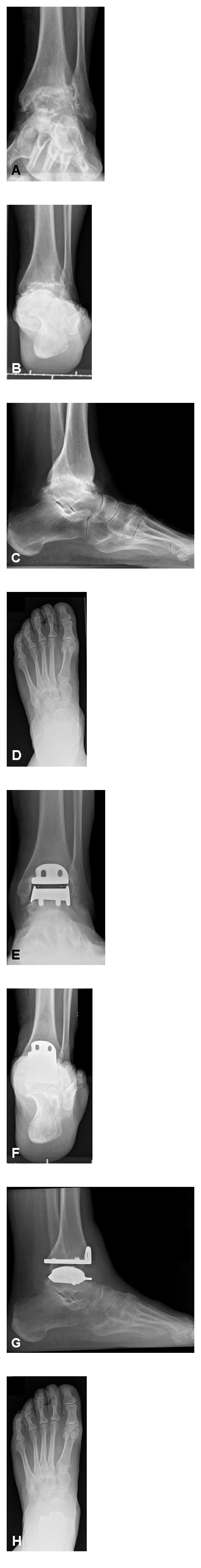
Radiologic evaluation preoperatively (A–D) and after 5 years (E–H): AP view of the ankle (A, E), Saltzman alignment view (B, F), lateral view (C, G), and AP view of the foot (D, H). Same patient as in Figure 1.

After surgery, the foot is protected by a splint. When the wound condition is proper, typically 2 to 4 days after surgery, the foot is placed in a short leg weight-bearing cast or a walker for 6 weeks, and a brace may be used for 4 to 6 additional weeks. Most importantly, the patient is allowed for full weight-bearing from the beginning, with only exception where additional surgeries do not allow it (e.g., correcting tibial osteotomies). After the cast is removed, the rehabilitation program was started, with gradual return to full activities as tolerated. Radiological controls are made 6 weeks, 4 months, and 12 months after surgery and then annually (Figure 2).

## 4. Results

In the orthopaedic literature, there are very few studies that compare implants head to head that are either Level I or Level II, and the superiority of an implant design over another cannot be supported by any available data from Level IV studies [42]. The experiences of several national joint registries have been published previously [43–46]. No statistically significant risk factors (e.g., age, gender, type of prosthesis, underlying etiology) have been identified as influencing survivorship of prosthesis components in Norwegian Arthroplasty Register [43], Finnish Arthroplasty Register [46], and New Zealand National Joint Registry [45]. In the Swedish Ankle Arthroplasty Register, lower age at TAR surgery was associated with increased risk of revision whereas preoperative diagnosis or gender did not [44]. Prosthesis misalignment and aseptic loosening have been consistently found to be the most common cause for prosthesis revision [43–46]. A recent systematic review of the literature including 13 Level IV studies with 1105 TARs showed the overall failure rate of approximately 10% at 5 years with a wide range between 0% and 32% [42].

Based on our own registry on 394 ankles (female, 199; male 195; mean age 59.7 [25.3–90.0] years) with a minimal followup of 5 years, our revision free rate at 5 years was 95.1% and 85.6% at 10 years. The revision rate was higher in posttraumatic osteoarthritis than in primary or systemic osteoarthritis. There was no difference in outcome between female and male patients. Over 60-year-old patients evidenced, fewer complications than those patients of less than 60 years old, and they had also fewer revisions.

## 5. Complications

**Figure 3:**
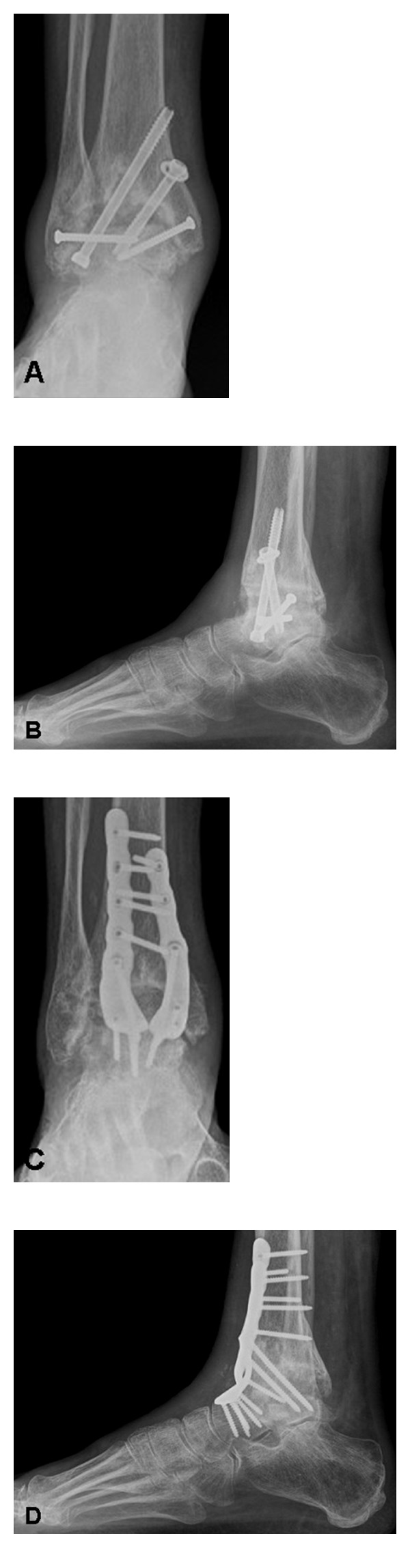
71-year-old male patient, 5 years after attempted fusion of the ankle (A-B). He was never pain-free. His orthopaedic surgeon led him in believe that the ankle was completely fused. One year after revision arthrodesis using a rigid anterior plate fixation, the fusion was healed and the patient was pain-free (C-D).

Numerous reports describing several techniques for ankle arthrodesis report the fusion rate of 85% or greater, which may depend on the presence of infection, deformity, avascular necrosis, and nonunion [12, 47]. However, only in few studies, a CT scan has been used to assess the postoperative osseous healin; therefore, the reported fusion rate of up to 100% may be overestimated. Since 2008, a total of 38 patients presented in author's outpatient clinic with persistent pain after ankle arthrodesis related to a nonunion (Figure 3), 31 patients (81.6%) were thought to have undergone successful ankle fusion by their treating orthopaedic surgeon.

The incidence of nonunion in ankle arthrodesis to a certain extent depends on the surgical technique used [12, 47]. Open procedures involve greater soft-tissue stripping than limited open or arthroscopic techniques. Poor bone quality as typically is the case in elderly patients remains a challenge for achieving primary stability for both external and internal fixation. Newer techniques with rigid plate fixation have shown superior results but may be associated with a higher risk of soft tissue complications or need for hardware removal due to discomfort [13, 48, 49].

Beside nonunion, mal-union of the fused ankle is another one of the most disabling conditions. The most common mal-union is due to unphysiological plantar flexed position [31]. In addition to consecutively developed metatarsalgia, the longstanding plantar flexed mal-union may be a risk factor for development of degenerative changes in subtalar and/or talonavicular joints. Fusion in dorsiflexion, by contrast, may lead to “back-kneeing” or genu recurvatum. This, in turn, places the patient's center of gravity in front of the weight-bearing axis causing vaulting over the improperly positioned foot. Varus or valgus malunion may also present problems but usually only if severe.

**Figure 4:**
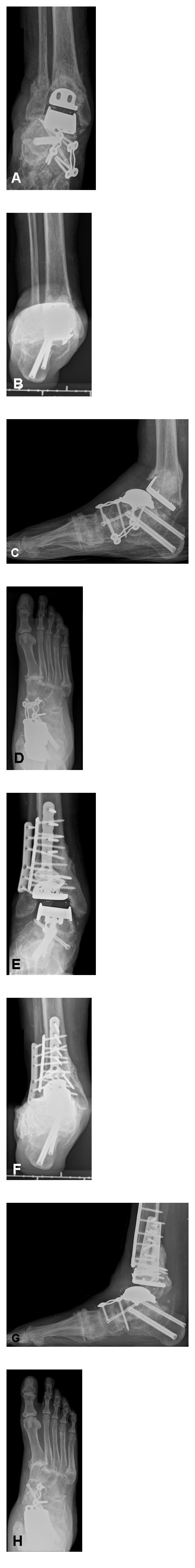
Periprosthetic fracture in a 61-year-old female patient with rheumatoid arthritis after struggling on the stairs. Marked angulation into valgus (A-B) and recurvatum (C), with supination of the foot (D). Uneventful evolution after internal fixation. One year afterwards, the ankle was stable and the patient was able to walk without any pain, though the ankle was still in slight valgus (E-F). Correct situation in lateral view (G) and AP view (H) of the foot.

In preserving joint motion, TAR offers an excellent alternative to arthrodesis and its sequelae [50–52]. The early complications after TAR include break down of wounds and superficial and deep infection [53, 54]. With current techniques and implants, the risk of primary loosening has dramatically decreased. Nevertheless, in the presence of poor bone quality, a successfully replaced ankle may be susceptible to periprothetic fracture during early remodelling phase (Figure 4).

The main risk of failure after total ankle replacement results from not achieving a balanced ankle joint complex [55]. As a majority part of end-stage osteoarthritic ankles will present with associated problems such as misaligned hindfoot, varus or valgus tilt of talus within the mortise, instability, or soft tissue contractures, the surgeon must be familiar with addressing these associated problems to get a successful replaced ankle [39, 56]. Surgeon's experience may thus play a superior role for success in TAR [57–60].

The use of TAR in elderly patients still remains controversial in orthopaedic surgery [52]. Kofoed and Lundberg-Jensen [61] have performed a prospective study reporting 100 consecutive cases of patients with osteoarthritis or rheumatoid arthritis with a followup up to 15 years. In all patients, Scandinavian Total Ankle Replacement has been used. All patients were divided into two groups: younger and older than 50 years. The authors found that TAR is a safe and reliable procedure for both, younger and elderly patients with 75.0% and 80.6% survivorship at 6 years, respectively [61]. Several other studies have shown a more favourable outcome of TAR in patients with rheumatoid arthritis and elderly low-demand patients with degeneration ankle arthritis [62–67]. Spirt et al. [68] have analyzed the cause and frequency of reoperation and failure after 306 primary total ankle arthroplasties using DePuy Agility prosthesis. Age at the time of the primary TAR was the only covariate that had an impact on the hazard of reoperation and failure: each one-year increase in age corresponded with a 1.9% relative decrease in the hazard of reoperation and 3.5% decrease in the hazard of failure [68].

The ideal patient for TAR continues to be debated within the orthopaedic foot and ankle surgeons [52]. However, in the most studies, the ideal candidate for TAR has been identified as reasonably mobile, *middle aged or older patient*, with no obesity or overweight and well aligned and stable hindfoot [52, 62, 69–76].

## 6. Conclusions

TAR has evolved as a reliable and safe alternative to arthrodesis in the treatment of end-stage ankle osteoarthritis [50, 53, 77]. Reduction in device constraint realized by the contemporary prosthetic designs in comparison with the first generation devices and improved instrumentation has markedly contributed to this higher success. Clinical longevity of TAR is dependent upon a correct balance between the intrinsic mobility allowed by the design and the presenting pathology of the patient [55]. This is further influenced by the ability of the surgeon to appropriately balance the soft tissue constraints and correctly align the components [57–60]. Despite improvement in designing appropriate surgical training, experience and technique will ultimately determine the results of total ankle arthroplasty [57–60].

The elderly patient may benefit more by TAR then the alternative ankle arthrodesis. First, postoperative rehabilitation after TAR is easier than that after ankle arthrodesis, allowing for full weight-bearing from the beginning. Immobilization and protection time is usually also markedly shorter for TAR. Thus, loss of articular and muscular function may be less than that after ankle arthrodesis [78–80]. Second, TAR may better restore hindfoot biomechanics, resulting in less gait adoptions and functional impairment [81]. Finally, TAR is in particular promising for elderly patients as the physical demands are, in general, lower. In summary, TAR has yielded to a valuable alternative to ankle arthrodesis and thus can be recommended in elderly patients as a very promising option to regain life quality and function.
